# Carbon Storage Patterns of *Caragana korshinskii* in Areas of Reduced Environmental Moisture on the Loess Plateau, China

**DOI:** 10.1038/srep28883

**Published:** 2016-07-14

**Authors:** Chunmei Gong, Juan Bai, Junhui Wang, Yulu Zhou, Tai Kang, Jiajia Wang, Congxia Hu, Hongbo Guo, Peilei Chen, Pei Xie, Yuanfeng Li

**Affiliations:** 1 College of Life Sciences, Northwest A&F University, No. 22 Xinong Road, Yangling, Shaanxi 712100, China; 2 College of Life Sciences, Zhejiang University, No. 866 Yuhangtang Road, Hangzhou, Zhejiang 310058, China

## Abstract

Precipitation patterns are influenced by climate change and profoundly alter the carbon sequestration potential of ecosystems. Carbon uptake by shrubbery alone accounts for approximately one-third of the total carbon sink; however, whether such uptake is altered by reduced precipitation is unclear. In this study, five experimental sites characterised by gradual reductions in precipitation from south to north across the Loess Plateau were used to evaluate the *Caragana korshinskii*’s functional and physiological features, particularly its carbon fixation capacity, as well as the relationships among these features. We found the improved net CO_2_ assimilation rates and inhibited transpiration at the north leaf were caused by lower canopy stomatal conductance, which enhanced the instantaneous water use efficiency and promoted plant biomass as well as carbon accumulation. Regional-scale precipitation reductions over a certain range triggered a distinct increase in the shrub’s organic carbon storage with an inevitable decrease in the soil’s organic carbon storage. Our results confirm *C. korshinskii* is the optimal dominant species for the reconstruction of fragile dryland ecosystems. The patterns of organic carbon storage associated with this shrub occurred mostly in the soil at wetter sites, and in the branches and leaves at drier sites across the arid and semi-arid region.

The global mean temperature is expected to continuously increase by an estimated 1.8 °C to 4.0 °C by the end of the 21^st^ century^1^, and global warming also causes precipitation levels and patterns to change in local ecosystems^2^,^3^. The global land area as a whole receives less-than-normal precipitation, although precipitation responses to El Niño events may differ in different regions^4^, thereby causing new drylands to develop in many areas across the globe. Drylands account for 58.5% of the world’s dryland area in Asia and Africa^5^. Piao *et al.* showed that precipitation is decreasing overall in the arid and semi-arid regions of China because of climate change^6^, and such areas are extending to the farming-pastoral transition zone as global warming continues to affect water availability for both natural ecosystems and human needs^7^,^8^.

Carbon sequestration capacity and ecosystem stability are known to be generally weak in dryland areas^9^,^10^, and global-scale drought conditions have severely decreased the total carbon in arid and semi-arid ecosystems worldwide^11^. Thus, reduced precipitation has a strong negative influence on carbon sequestration^12^,^13^ because water and carbon are tightly associated in ecosystems^14^,^15^. Therefore, many researchers have focused their studies on the relationship between plant and ecosystem functional features and rainfall. Such studies have shown, for example, that the photosynthesis rate, productivity, and individual size of many plant species decline as drought stress increases^16^,^17^,^18^,^19^. Plant population density and coverage also decrease because of drought, which results in decreased plant and soil carbon sequestration^20^.

It is feasible to define and adopt reasonable measures to minimise the negative effects of climate change in arid and semi-arid ecosystems, although decreased carbon fixation capabilities and other ecological functions pose serious challenges. Selecting a suitable xeric species to cultivate and absorb the increased levels of CO_2_ from the air, for example, could help maintain ecosystem stability under drought conditions. Enhanced regrowth management is currently being used to protect biodiversity and manage carbon sequestration in ecosystems affected by climate change^21^. Afforestation is also an effective strategy for promoting soil carbon sequestration in semi-arid regions. In Central China from 1980 to 2010, this strategy was used to decrease soil disturbances, to reduce carbon release^22^, and/or to increase soil organic matter^23^.

The Grain-for-Green programme was the first and most ambitious of several Chinese “payment-for-ecosystem-services” initiatives, and it began in the 1990s and was targeted towards arid and semi-arid parts of the country. Compared with the conditions in the early 1980s, sparsely vegetated areas declined by 18.1% and vegetated areas increased by 3.5% by the late 1990s in the Loess Plateau^24^. Approximately 26,867 million ha of reconstructed vegetation resulting from the Grain-for-Green project was expected to form a carbon pool by the end of 2008^25^,^26^ and it became an important strategy for increasing soil and plant organic carbon^8^,^23^. The general positive effects of vegetation restoration on soil organic carbon content (SOC) have been demonstrated at the patch, hill slope, and catchment scale in the Loess Plateau^25^. Forbidding agriculture and grazing has been associated with decreased desertification in Northwest China, which demonstrates that this approach is potentially effective for ecological restoration^27^. Compared with the SOC of cropland, the SOC in the surface soil of artificial shrub land in the Loess Plateau increased from 122% to 163% after 1976^28^.

Drought tolerance and species dominance are typically addressed when evaluating suitable species for reforestation and carbon fixation in arid areas^22^,^29^. In the Loess Plateau, dominant species can mitigate negative affect caused by reduced precipitation by preserving water. *Caragana korshinskii*, a drought-tolerant mesquite, is the species most often employed for vegetation restoration because it only slightly influences growth and carbon fixation in arid afforested areas within a certain range. Thus, *C. korshinskii, R. pseudoacacia, Pinus tabuliformis,* and *Hippophae rhamnoides* have been used for vegetation restoration in the Loess Plateau, and *C. korshinskii* and *R. pseudoacacia* have shown the most success^30^. In fact, afforested *R. pseudoacacia* planted 20–30 years ago has contributed significantly to the plant organic carbon density in the Loess Plateau^31^. It is estimated that the carbon uptake by shrub lands alone accounts for 30% of the total carbon sink in this area^32^,^33^, where carbon sequestration is particularly important because of the current climate warming trend^34^. The morphology, anatomy, and xerophilous physiological features of *C. korshinskii* widely distributed in the dryland of the Loess Plateau are also notable^35^; however, relatively few studies have attempted to explain why only certain species affect dryland plant and soil carbon densities^10^, particularly at mid- and high altitudes, where the frequency and severity of drought are projected to increase^36^. Currently, changes to plant growth and carbon sequestration under the prevailing precipitation gradient in these areas are not well understood. Thus, studying the physiological mechanisms underlying the positive growth and carbon fixation patterns of the dominant shrubbery in the Loess Plateau is highly worthwhile.

In the semi-arid central region of China (and throughout Central Asia in general) there is a natural precipitation gradient associated with latitude across the Loess Plateau^37^. The Loess Plateau covers 630,000 km^2^ of northwestern China, and it is a main target area for drought and soil erosion^38^ and an excellent example of the natural reduced precipitation gradient; thus, it provides a valuable opportunity to investigate the growth and carbon sequestration of *C. korshinskii* under drought increase as well as the effect of leaf functional and physiological traits on carbon fixation. The primary goals of the present study were to 1) determine the response of leaf ecological functional traits of *C. korshinskii* under reduced rainfall, 2) explore the physiological mechanism underlying carbon fixation of *C. korshinskii* under more harsh drought conditions, and 3) evaluate the carbon sequestration patterns of *C. korshinskii* under increasing abiotic environmental stress in the arid and semi-arid regions of China.

## Results

### Variation of Leaf Functional physiological Characteristics with Drought

**Figure 1:**
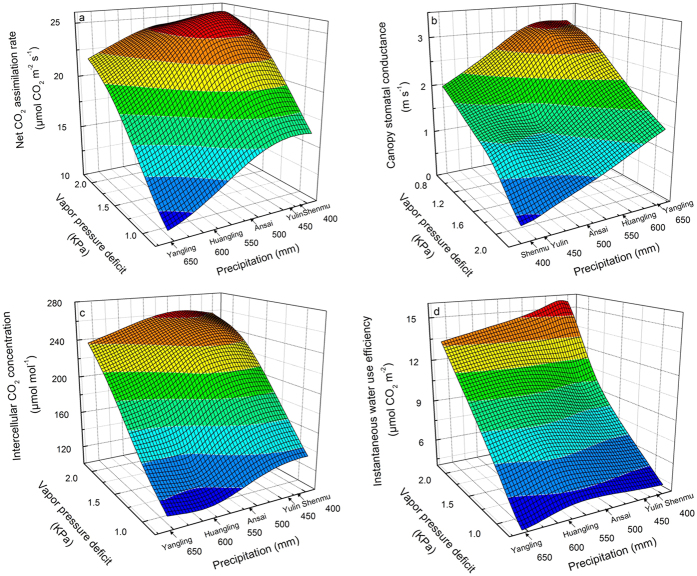
Three-way trait relationships of the leaf traits of *C. korshinskii*. (**a**) Net CO_2_ assimilation rate (*A*_n_), (**b**) canopy stomatal conductance (*g*_c_), (**c**) intercellular CO_2_ concentration (*C*_i_), and (**d**) instantaneous water-use efficiency (*WUE*_i_) versus precipitation and VPD at the five experimental sites.

Gas exchange parameters are among the main leaf functional characteristics of *C. korshinskii*, and they co-vary with precipitation and leaf-air vapour pressure deficits (VPD) at the five experimental sites as shown in Fig. 1. The leaf net CO_2_ assimilation rate (*A*_n_), intercellular CO_2_ concentration (*C*_i_) and instantaneous water-use efficiency (*WUE*_i_) presented similar trends in response to reduced precipitation and two VPD levels (Fig. 1). At each site, the *A*_n_, *C*_i_ and *WUE*_i_ were always enhanced with higher VPD than with lower VPD (Fig. 1a,c,d). Compared with the conditions in Yangling, the *A*_n_ in Huangling, Ansai, and Yulin increased gradually and remained at a relatively high level, although a slight decrease was observed in Shenmu under reduced precipitation (Fig. 1a). Notably, the leaf *A*_n_ increased by approximately 50% in Ansai, which received 514.8 mm precipitation, compared with that in Huangling, which received 578.7 mm precipitation under higher VPD. In addition, the *C*_i_ at five sites with gradually reduced precipitation continued to increase (Fig. 1c). A similar trend was also observed in the *WUE*_i_, especially in the driest area Shenmu, which presented a *WUE*_i_ that was significantly higher than that of the other four sites, even under higher VPD (Fig. 1d). The change in canopy stomatal conductance (*g*_c_) was opposite that of the above parameters, and it gradually decreased with less precipitation and increased VPD (Fig. 1b); however, the decreased *g*_c_ did not affect the *C*_i_ intake.

### Changes in physiecological features with Increasing Drought

**Figure 2:**
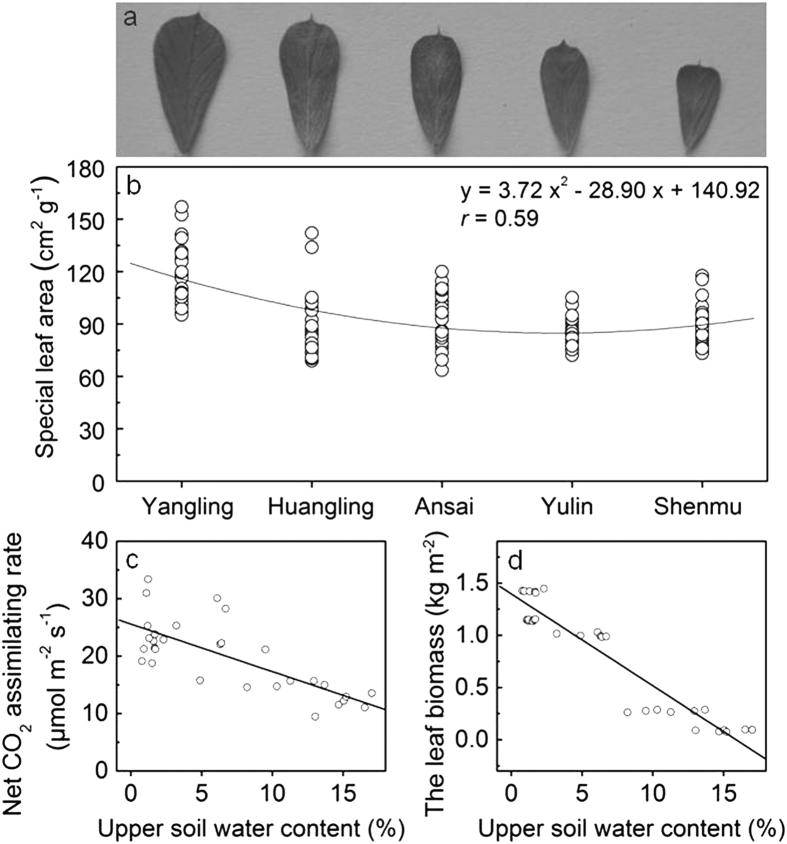
(**a**) Leaf area change at the five sites under drought conditions. (**b**) Specific leaf area (SLA) decreased; (**c**) net CO_2_ assimilation rate (*A*_n_) increased significantly (y = −0.8271x + 25.5981, *R* = −0.75), and (**d**) leaf biomass increased rapidly (y = −0.0879x + 1.3964, *R* = −0.95) (*P* < 0.0001) in *Caragana korshinskii* leaves at the five experimental sites as the upper soil water content decreased.

Compared with the *A*_n_ change trend, the spatial leaf areas (SLA) gradually decreased as the precipitation decreased across the five test sites. The SLA value at Yangling was the largest and significantly higher than that at the other four sites (Fig. 2a,b). The *g*_c_ values exhibited similar a trend as that of the SLA, in which the values at Yangling and Huangling were significantly higher than those at the other three sites (Fig. 1b), which indicated that a smaller leaf area with less precipitation was insufficient space for water loss but accumulates biomass and produces a thicker leaf. Additionally, the *g*_c_ values seem to be more sensitive than the SLA to water deficits (Figs 1b and 2b).

### Leaf biomass likely reflects a plant’s growth rate and annual net productivity

Both the *A*_n_ and leaf biomass of *C. korshinskii* increased sharply as the water content of the upper 100 cm soil layer decreased (Fig. 2c,d). Accordingly, the individual growth rate of *C. korshinskii* also increased with drought aggravation.

The biomass values of four plant organs (branches, leaves, rachis, and roots) were also determined to obtain the total plant biomass (PB), which significantly increased as the precipitation decreased at the five sites (Table 1) and was significantly correlated among the sites (*R* = −0.923, *P* < 0.05) (see Supplementary Table S1). The individual leaf and root biomasses also significantly increased and were negatively associated with annual precipitation (AP) (*R* = −0.970, *P* < 0.05). The *A*_n_ was enhanced by water availability as reflected by a positive relationship between plant biomass and reduced precipitation.

**Figure 3:**
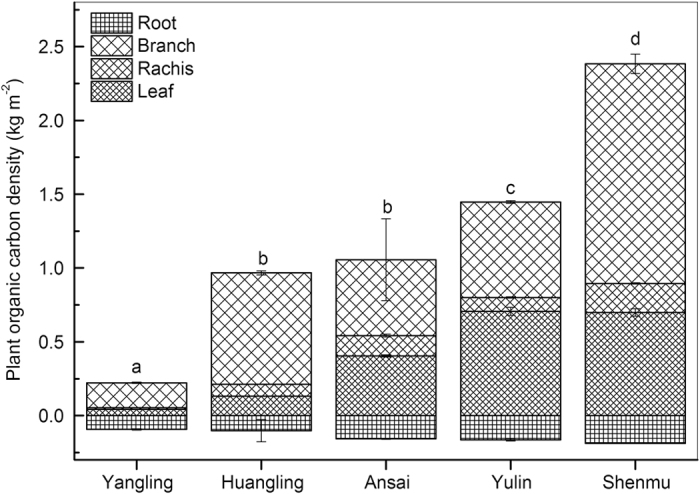
Plant organic carbon density in the plant organs of *Caragana korshinskii* with reduced precipitation at the five sites. Leaf, root, and total PCOD increased rapidly as precipitation decreased. Values are the mean ± se. Letters indicate significant differences between sites (*P* < 0.05).

The plant organic carbon density (POCD) naturally showed the same tendency as the total plant biomass.As the precipitation decreased, the POCD in all plant organs increased, with branches accounting for the largest proportion (58%) in Shenmu, followed by the leaf (44% in Yulin and 27% in Shenmu) and root (approximately 7% in Shenmu) as shown in Fig. 3.

### Vertical Distribution of SOC among the Five Sites

The soil moisture content (SMC) in the 0–50 cm soil was higher at the Yangling, Huangling, and Ansai sites and lower at the Yulin and Shenmu sites, thus reflecting the influences of reduced precipitation on SMC (see Supplementary Fig. S1). However, the water content in the 50–100 cm soil layer fluctuated to some extent. The SMC in the layers below 100 cm, conversely, were stable at all sites, although differences were observed at sites that were subjected to different amounts of precipitation.

**Figure 4:**
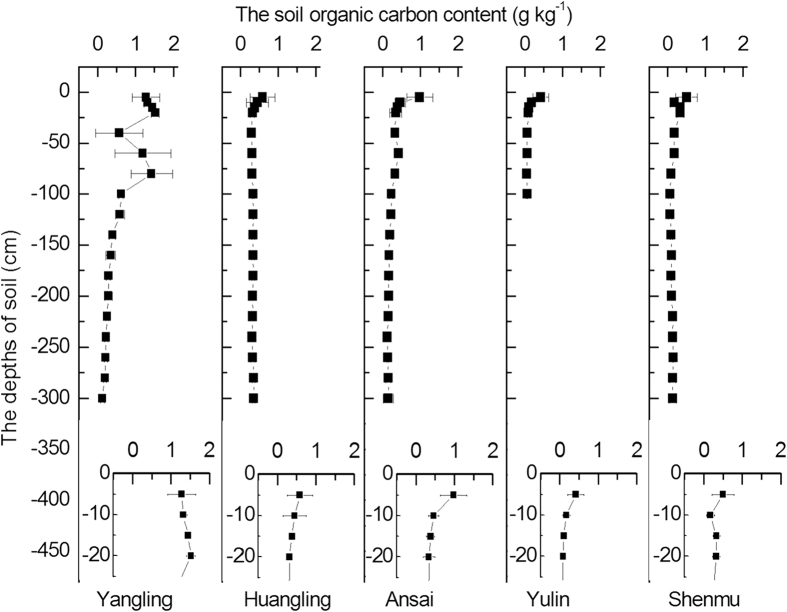
Vertical distribution of the soil organic carbon content with the soil depth and variations among the five experimental sites with reduced precipitation.

**Figure 5:**
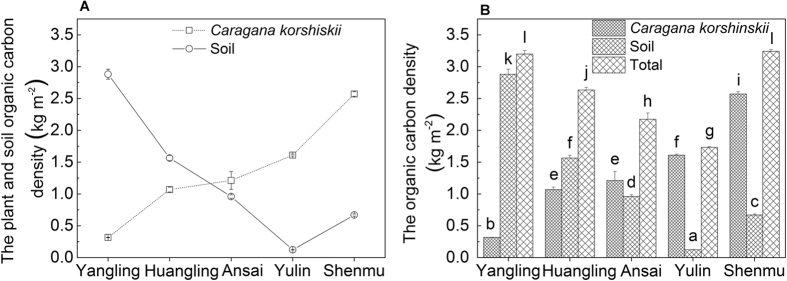
(**A**) Plant organic carbon density of four organs of *Caragana korshinskii* and in soil that was collected from the five experimental sites with reduced precipitation. Values are the mean ± se. (**B**) Variations in the plant, soil, and total organic carbon density at the five sites, which ranged from wet to dry. Letters indicate significant differences between sites (*P* < 0.05).

SOC decreased gradually with soil deeper at each site and was particularly significant in the 0–20 cm layer (Yangling was the only exception to this rule.) When the soil depth exceeded 100 cm, the SOC was relatively stable (Fig. 4). In the 0–20 cm layer, the SOC accounted for 30%, 25%, 20%, 15%, and 12% of the total SOC in the 0–300 cm soil profile at Yangling, Huangling, Ansai, Yulin, and Shenmu, respectively (Fig. 4). The total SOC of the observed soil profile in Yangling was greater than that of the other sites. The soil organic carbon density (SOCD) showed a sharp decrease from Yangling to Huangling and then decreased significantly in Ansai and Shenmu in the 300 cm soil profile and in the 100 cm soil profile in Yulin (Fig. 5). Compared with the SOCD at Yangling, the soil under the areas forested with *C. korshinskii* in Huangling, Ansai, and Shenmu showed 60%, 50%, and 20% decreases of SOCD within the 0–300 cm (40% decrease of SOCD within 0–100 cm in Yulin) layer, respectively.

### Plant and SOCD among the Five Sites

Statistical analyses of the changes in the organic carbon density of the afforested *C. korshinskii* and soil at our five test sites demonstrated clear and consistent trends. The organic carbon density of *C. korshinskii* increased gradually as the precipitation decreased (Fig. 5A). Compared with that of the Yangling site, the POCD increased significantly to 0.75 kg m^−2^ in Huangling, to 0.89 kg m^−2^ in Ansai, to 1.29 kg m^−2^ in Yulin, and to 2.25 kg m^−2^ in Shenmu (Fig. 5B, Table 2). A negative and significant correlation was observed between POCD and AP (*R* = −0.915, *P* < 0.05) (see Supplementary Table S1). The SOCD decreased gradually as the precipitation decreased; however, the SOCD in Yulin was lower than in Shenmu because soil samples just collected from 0–100 cm soil layers in Yulin (Fig. 5A). The SOCD also showed a high correlation with the AP (*R* = 0.955, *P* < 0.01) (see Supplementary Table S1). The POCD level began to shift and surpassed the SOCD from Ansai onward, and the most remarkable difference between the POCD and SOCD appeared in Shenmu. Therefore, the total organic carbon density (TOCD) distribution along the reduced precipitation was remarkably higher in wetter Yangling than in drier Ansai and Yulin (*P* < 0.05), whereas it was enhanced in Shenmu, even beyond that of Yangling. This phenomenon was likely a result of the contribution of the POCD (79.35%) because the SOCD was relatively low (20.65%) in Shenmu (Table 2).

The relative contribution of the soil to the TOCD decreased, whereas the contribution of plants to the TOCD increased with the reduced precipitation along the five sites (Table 2). The AP was approximately 653 mm in Yangling, which had 10% POCD to TOCD, whereas the AP was approximately 419 mm in Shenmu, which had 79% POCD to TOCD. Organic carbon was stored initially in the wetter soil, then in the roots in drier areas, and in the leaves and branches in the much drier areas in Shenmu. Only a small proportion of the organic carbon was stored in the rachis at the five sites (Figs 3 and 5B).

**Figure 6:**
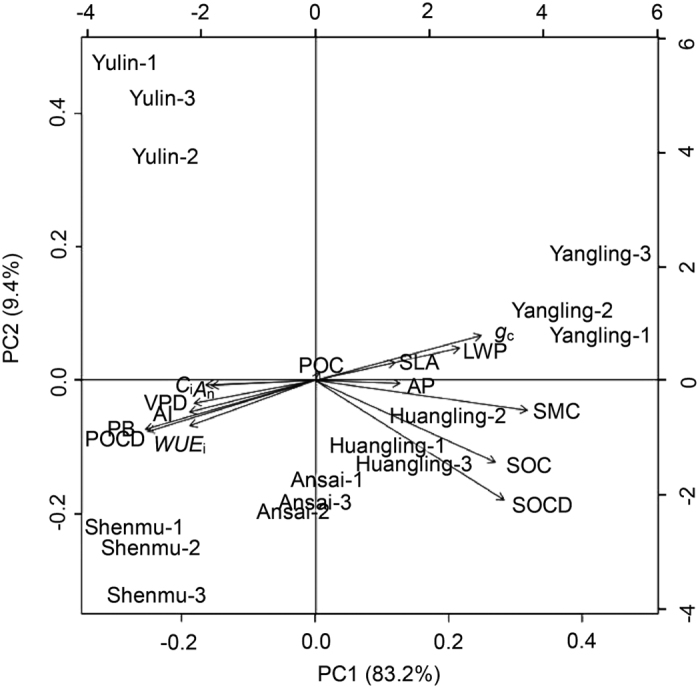
Biplot of the relative correlation between the environmental factors and functional traits of *Caragana korshinskii* at the different sites. PCA ordination diagram for 25 variables, including environmental conditions (annual precipitation, soil moisture content, and leaf-air vapour pressure deficit), carbon fixation vegetation characteristics (special leaf areas, leaf water potential, net CO_2_ assimilation rate, canopy stomata conductance, intercellular CO_2_ concentration, plant biomass, instantaneous water use efficiency, plant organic carbon, and plant organic carbon density) and physical properties of the soil (soil moisture content, soil organic carbon, and soil organic carbon density) detected at the five experimental sites with reduced annual precipitation. Note: The first two PCA axes explain 92.6% of the total variability. The fifteen best-fitting characteristics are shown.

Relationship between physiological characteristics and environmental factors is worth further attention. Variations in the physiological and functional traits of *C. korshinskii* and the environment factors were analysed in the five sites as shown in Fig. 6. As shown in Fig. 6 and Supplementary Fig. S2, the PCA provided an overview of these data. The first principal component (PC1) explained 83.2% of the total variation, whereas the second component (PC2) explained 9.4%. Three biological replicates of every experimental site were assembled and then separated from each other so that favourable repeatability could be maintained in identical experimental areas despite changes in the parameters. The PCA biplot shows the relative correlation between the environmental factors and functional traits of *C. korshinskii* at the different sites and indicates the 15 best-fitting characteristics (Fig. 6). The AP changes were consistent with the varying environment factors, such as the aridity index (AI), VPD, and SMC. Pearson correlation analysis was also performed for 14 variables to further investigate the correlation between these parameters at the five sites along the reduced precipitation as shown in Supplementary Table S1, including the environmental conditions (AP, AI, SMC and VPD), vegetation carbon fixation characteristics (SLA, leaf water potential (LWP), *A*_n_, *g*_c_, *C*_i_, PB, *WUE*_i_ and POCD) and physical properties of the soil (SOC and SOCD). A highly significant and positive correlation was identified between the POCD and AI (*R* = 0.971, *P* < 0.01) and between the POCD and VPD (*R* = 0.961, *P* < 0.01) (see Supplementary Table S1).

Higher SMC values were observed because of the relatively abundant AP in Yangling and Huangling as well as the higher LWP, SLA, and soil carbon fixation capacity (Fig. 6). However, the POCD, one of the positive indicators of plant carbon sequestration, was dominant in Shenmu according to the PCA, and it was highly correlated to *C*_i_, *WUE*_i_ and PB (Fig. 6), which is consistent with the information shown in Supplementary Table S1. A highly significant and positive correlation was found between the POCD and *C*_i_ (*R* = 0.855, *P* < 0.05), POCD and *WUE*_i_ (*R* = 0.989, *P* < 0.01), and POCD and PB (*R* = 1, *P* < 0.01). The POCD in Shenmu was highly negative correlated to the SMC, AP, and LWP (Fig. 6). A highly significant and negative correlation was found between the POCD and SMC (*R* = −0.888, *P* < 0.05), POCD and AP (*R* = −0.915, *P* < 0.05), and POCD and LWP (*R* = −0.907, *P* < 0.05) (see Supplementary Table S1). Conversely, a highly significant negative correlation was observed between the AP and *C*_i_ (*R* = −0.963, *P* < 0.01) and between the AP and PB (*R* = −0.923, *P* < 0.05), although a significant positive correlation was observed between the AP and *g*_c_ (*R* = 0.982, *P* < 0.01) as well between the AP and SLA (*R* = 0.862, *P* < 0.05) (see Supplementary Table S1). The SLA was extremely significantly correlated with the LWP (*R* = 0.943, *P* < 0.01), and the LWP was significantly correlated with the AP (*R* = 0.968, *P* < 0.01) (see Supplementary Table S1).

Furthermore, the *A*_n_, which is a physiological characteristic related to carbon fixation, was significantly correlated with the functional traits *C*_i_ (*R* = 0.894, *P* < 0.05) and *g*_c_ (*R* = −0.905, *P* < 0.05) (see Supplementary Table S1). The *A*_n_ was strongly related to the drought factor SMC (*R* = −0.917, *P* < 0.05). Physiological indices, such as the *A*_n_ and *C*_i_, also exhibited responses similar to those of the PB and POCD, whereas the SLA and *g*_c_ showed the opposite effects (Fig. 6). These phenomena indicate that carbon sequestration was subject to external environmental factors, higher VPD easing to force air intake accelerated *C*_i_ diffusion in smaller leaf under less precipitation, even improving the *A*_n_. The improved *A*_n_ and inhibited transpiration (*T*_r_) caused by the lower *g*_c_ from drier air and soil together enhanced the *WUE*_i_, which increased the PB and thereby increased the POCD (see Supplementary Fig. S3).

## Discussion

### Physiological and Leaf Functional Traits Involved in Increasing the POCD

In general, increasing precipitation can promote plant growth in temperate biomes, although it occurs at the expense of decreased vegetation production (which is particularly significant for cold, deciduous coniferous forests)^39^. Although water stress has been shown to slow growth and photosynthesis, this evidence is primarily derived from brief studies that do not account for all longer-term acclimation processes that are relevant in tree species.

In general, the total tree biomass increased with increasing precipitation throughout most of China^40^. In this study, certain physiological indices, such as *A*_n_, *C*_i_, and *WUE*_i_, as well as PB and POCD increased as the precipitation decreased. The acclimation to long-term water stress leads to higher mesophyll conductance contributes to higher *WUE*_i_, which moderates constraints on *A*_n_ and reduces leaf oxidative stress^41^. Shrubs thereby accelerating *C*_i_ diffusion among mesophyll and increasing their *WUE*_i_ to improve their biomass and relieve the stress. WUE promotes the most important plant development stages: increased stomata sensitivity to CO_2_ and light (which enhances the plant’s ability to respond to environmental changes through lower stomatal conductance)^42^, which could explain why the decreased *g*_c_ did not affect the *C*_i_ intake in *C. korshinskii. WUE* also promotes another most important plant development stages: changes in the structure and mode of the vascular tissue to promote an accumulation biomass, which allows the plant to more effectively use its environmental conditions to survive^43^. That’s why the biomass of the shrub branches of *C. korshinskii* is much larger than that of the leaves. Thus, when AP reduction occurs, the shrub responds by increasing the number of branches relative to the number of leaves. This phenomenon suggests that plant conducting tissue becomes more developed under reduced precipitation^44^. Recent research has shown that *C. korshinskii* can accumulate more carbohydrates in its stems and branches in response to extreme drought stress, and such changes are necessary for new leaves to re-sprout and re-grow^45^.

Photosynthesis is exceptionally sensitive to water stress^46^. The leaf is the most important photosynthetic organ, and certain indices are the ideal indicators for carbon sequestration in stressed environments. SLA is one such trait in the leaf-economics spectrum, and it is an especially easy index to measure and can be readily determined for numerous samples^47^. Our results suggest that the SLA decreased when *C. korshinskii* suffered the LWP caused by reduced precipitation, whereas the leaf thickness simultaneously increased. Changes in leaf shape may have helped *C. korshinskii* reduce transpiration, improve the water supply, and accelerate *C*_i_ diffusion because of decreasing mesophyll^40^.

We attributed the increase in plant carbon storage to increased PB and POCD because of the enhanced *A*_n_ and *WUE*_i_ which was determined by the plant physiological parameters *C*_i_ and *g*_c_ and the functional trait SLA as the plant and soil responded to AP reduction. The SMC was correlated with all the observed physiological characteristics as well as six environment factors. Our results suggest that reduced precipitation triggered the protected functional response (e.g. *g*_c_) and positive physiological response (e.g. *C*_i_, *A*_n_ and *WUE*_i_) and benefited plant carbon fixation.

### SOCD Degradation and Reduced Precipitation

Understanding the distribution of organic carbon storage in soil profiles is crucial for assessing regional, continental, and global SOC stores and predicting the consequences of global climate change^48^. SOCD can be enhanced artificially by reconstructing vegetation in arid and semi-arid areas^8^. The shrublands in the Loess Plateau are the largest contributors to SOC according to an analysis of land-use conversions on SOC sequestration^28^. In this study, we determined the effects of reduced precipitation on the SOC in the 0–300 cm profile at five sites, and our results showed that soil organic carbon storage accounted for a larger proportion in wetter areas (Yangling) and a smaller proportion in drier areas (Shenmu) of the TOCD. This pattern may be attributable to the input of aboveground plant parts and the decomposition of underground litter, which both affect soil organic carbon storage^49^. Published studies have focused mostly on the topsoil (0–100 mm)^50^ although deeper soil layers also form a sink for carbon sequestration^51^. Our results show that the SOC and SOCD both decreased in accordance with reductions in AP and SMC (which is consistent with the results of Saiz *et al.*^52^). The amount of precipitation has a profound effect on the chemical properties of soil and the nutrient status of forest soils because it accelerates rock weathering and basic cation and nitrate leaching^53^.

The *C. korshinskii* carbon sequestration model under drought stress that was established in this study reflects the clear relationship between functional traits and environment factors. LWP was most sensitive to AP, VPD, and SMC. AP reductions caused higher VPDs and lower SMCs, which then caused lower LWP, thereby resulting in smaller SLA and *g*_c_ values. Both reduced SLA resulting in decreased mesophyll and higher VPD easing to force air intake accelerated *C*_i_ diffusion, although *g*_c_ decreased under less precipitation. The *A*_n_ is subsequently enhanced, and both the improved *A*_n_ and inhibited *T*_r_ caused by lower *g*_c_ with drier air and soil enhanced the *WUE*_i_. Then led to an increase in total PB, which was attributed to the *WUE*_i_ and *A*_n_. Ultimately, the plant then showed an enhanced POCD and strengthened carbon sequestration capacity.

### Management Implications

A considerable proportion of land in our study area was severely affected by soil erosion and desertification prior to the enactment of policies to transform farmlands back into forests. As a method of mitigating climate change, the promotion of forestry activities, such as artificial carbon sequestration, can reduce the concentration of carbon dioxide in the atmosphere and allow the forest carbon sink to function effectively. Meyer^54^ and Yang *et al.*^10^ demonstrated that shrubs in cold deserts can store large amounts of carbon in their biomass and the surrounding soil. Forestry is an important method of responding to climate change because it increases the carbon sink by increasing and protecting shrub vegetation; however, it is also a potential method of accelerating necessary ecological construction and promoting sustainable development in general. Our experiments confirmed that positive interactions occurred between the xeric shrubbery and drier environments, including increased shrub biomass and enhanced carbon sequestration capacity. Our results implicate that drought stress leads to significantly distinct organic carbon storage patterns in the plant population in arid and semi-arid regions, with carbon stored at higher proportions in the soil in wetter areas (Yangling), in the roots in drier areas, and in the leaves and branches in the driest areas (Shenmu). Reduced precipitation shaped and activated the functional and physiological characteristics of drought tolerance in the shrub layer and benefited the carbon fixation and organic carbon storage patterns throughout the plant community.

In summary, the protective response of canopy stomatal conductance to atmosphere drought and the positive response of gas exchange parameters to physiological drought are all the defenses of *C. korshinskii* to drought. The species is an optimal species for reconstructing ecosystems in arid and semi-arid regions and sequestering CO_2_ from the atmosphere to mitigate global climate change. Future research should focus on determining the AP threshold for biomass and carbon sequestration capacity reductions and testing the feasibility and outcomes of cultivating of this species in arid and semi-arid areas worldwide.

## Conclusions

Our results showed that carbon sequestration is subject to external environmental water stress caused by reduced precipitation. The higher VPD and smaller SLA values accelerated *C*_i_ diffusion under reduced precipitation and subsequently enhanced the *A*_n_. The improved *A*_n_ and inhibited *T*_r_ caused by the lower *g*_c_ with drier air and soil enhanced the *WUE*_i_, which increased the PB and thereby increased the POCD. The relative contribution of the plants to the TOCD increased from 10% to 79%, whereas the relative contribution of the soil to the TOCD decreased from 90% to 21% across the five sites along the wet to dry gradient. Thus, the TOCD in the wetter Yangling was almost the same as that of the much drier Shenmu. *A*_n_ increase in the POCD of *C. korshinskii* was triggered by reduced precipitation over a certain range, whereas a decrease in the SOCD was inevitable. Our results also suggest that reduced precipitation results in significantly distinct organic carbon storage patterns in the shrub layer, with more of carbon primarily stored in the soil in wetter areas (Yangling), and in the leaves and branches in drier areas (Yulin and Shenmu) of the arid and semi-arid Loess Plateau regions.

## Materials and Methods

### Experimental Sites

The Loess Plateau of northwestern China exhibits several distinct characteristics, including the most severe water shortages and soil erosion in the world^55^. This area has exhibited frequent drought extremes from 1971 to 2010^56^. *C. korshinskii* is the dominant vegetative species of the Loess Plateau and distributed throughout the region, and it provides key ecosystem-related functions in this severe environment. To clarify the contribution of this shrub to carbon sequestration, five experimental sites on the Loess Plateau with decreased precipitation were selected from south to north as shown in Supplementary Fig. S4. The annual precipitation and transpiration data was acquired from Ecological Environment Database of Loess Plateau (http://www.loess.csdb.cn/pdmp/index.action). The AI was then calculated according to Deng *et al.*^20^. with annual precipitation and transpiration.

The *C. korshinskii* population has been planted as an artificial ecosystem management strategy for approximately two decades at each site, and grazing is not practised at these sites. *C. korshinskii* coverage is located in flat areas, including farmland and mountain bases, and planted in several rows and columns. The column spacing and row spacing are 2 m × 2 m, and the density is approximately 0.45 N m^−2^. The list of geographical parameters and meteorological profiles of each test site can be found as Supplementary Table S2.

### Leaf Function Traits

The SLA, which represents the light-capturing surface built by the plant per unit investment of dry mass, is an indirect measure of the return on investment of the productive organ. The *C. korshinskii* leaf area was determined using a LI-3100 leaf area meter (LI-COR, Lincoln, NE, USA) from three individual plants with 6–9 leaves at each experimental site. Each fresh leaf was then dried at 65 °C for 2 days and weighed, and the leaf dry weight (LDW) and leaf area (LA) measurements were used to calculate the SLA as follows:


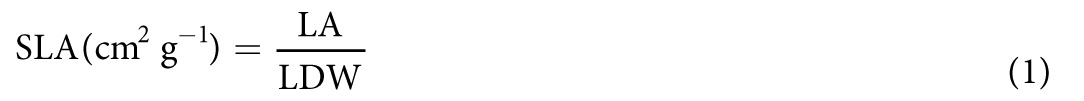


The *g*_c_ was calculated based on the sap flux estimates through a simplified form of the Penman-Monteith equation^57^:


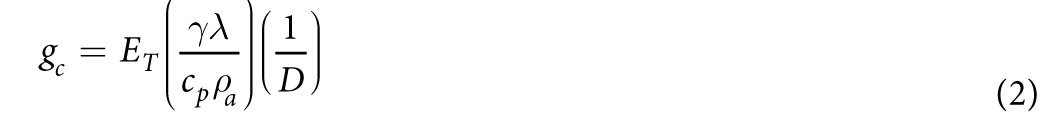


where *g*_*c*_ is the canopy stomatal conductance (m s^−1^), E_*T*_ is the canopy transpiration expressed on a ground area basis (kg m^−2^ s^−1^), *γ* is the psychrometric constant (Pa K^−1^), *λ* is the latent heat of water vaporisation (J kg^−1^), *c*_*p*_ is the specific heat of air (J kg^−1^ K^−1^), *ρ*_*a*_ is the density of dry air (kg m^−3^), and D is the saturated vapour pressure deficit of air (Pa).

Three mature and well-grown *C. korshinskii* individuals were selected from each shrub area. The gas exchange parameters of the leaves (e.g., *A*_n_, and *C*_i_) were measured from the leaves of three individuals at three aspects using a Li-6400 photosynthetic system (LI-COR Biosciences, Nebraska, USA) on sunny mornings in July and August 2010–2011 when the leaf temperature was approximately 33 °C. Meanwhile the VPD was obtained directly from the Li-6400 photosynthetic system. The *WUE*_i_ was then calculated according to Gong *et al.*^44^. All leaf parameters were measured in triplicate, and the actual leaf area was corrected as necessary.

### Plant Sampling and Biomass Measurement

Three 10 m × 10 m research quadrats were established to determine the height and diameter ranges of *C. korshinskii* along each experimental site on the precipitation gradient, and this information was then used to calculate the average shrub height and branch diameter. The average sample tree determination of three healthy and mature individual plants, which were selected according to the average shrub height and branch diameter, was performed to measure the *C. korshinskii* biomass. The width of the live crown of each sample shrub was measured, and then the shrub was felled, and its height and branch diameter were measured. Fresh masses of branches, leaves and other plant parts were measured, and then the root systems were dug up and weighed in the field. A portion of the plant organs were sampled, and the dry weight of the branches, leaves, rachis, and roots was determined. The biomass of each organic component was calculated according to Deng *et al.*^20^ and Li *et al.*^51^.

### Soil Sampling and Organic Carbon Content Measurement

Soil samples were separately collected from the area under three individual plants from the *C. korshinskii* population at each experimental site from late July to early August 2010–2011 using a cylindrical steel corer (diameter 8 cm, height 20 cm), and three replications were performed. When collecting the soil cores from the Huangling and Ansai sites, the soil depth of 0–320 cm was divided into 16 layers, and every 5 cm interval within 20 cm was considered a layer, and every 20 cm interval from 20 cm to 360 cm was considered a layer. For the other three sites, the sampling soil depths were all 320 cm except for that at Yulin, which was 100 cm. The corresponding soil water content was then measured gravimetrically^58^. The soils from each layer of each individual area were mixed into a composite sample using a cutting ring, and the soil bulk density was determined by the cutting ring method.

After removing litter and rock debris, the composite soil samples were air dried in an oven and then filtered through a 2 mm sieve. To determine the organic carbon content, the dry soil and plant organ samples (branches, leaves, rachis, and roots) were placed in a muffle furnace at 500 °C for 12 h for the complete combustion of organic compounds and then analysed using the flash combustion technique^59^ in a CHNS-O Elemental Analyser (Fisons Instrument, CA, USA).

### Plant and Soil Organic Carbon Density Calculation

The POCD including aboveground organic carbon density (AOCD) and root organic carbon density (ROCD), and SOCD were calculated using the method of Wang *et al.*^31^. The sum of the ROCD and AOCD is the POCD, and the sum of the POCD and SOCD is the TOCD.

The AOCD was calculated as follows:


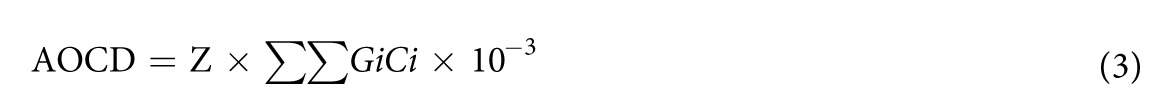


where AOCD is the organic carbon density of the aboveground parts including branches, leaves and rachis, *Z* is the average number of *C. korshinskii* individuals (N m^−2^), *Gi* is the biomass of different aboveground parts, and *Ci* is the organic carbon content of different aboveground parts.

The ROCD was calculated as follows:


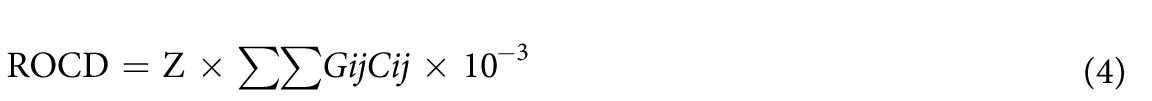


where ROCD is the root organic carbon density, *Z* is the average number of *C. korshinskii* individuals, *Gij* is the root biomass at the *i*^th^ layer and the *j*^th^ class (kg), and *Cij* is the root organic carbon content at the *i*^th^ layer (g kg^−1^).

The SOCD was calculated as follows:


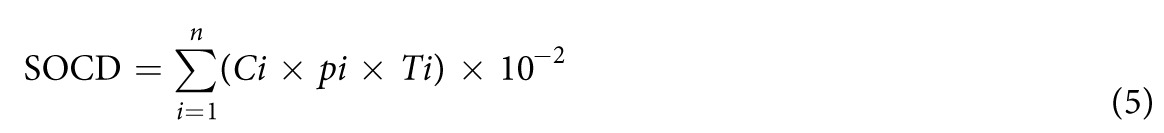


where SOCD is the soil organic carbon density at different soil depths (kg m^−2^), *Ci* is the soil organic carbon content at the *i*^th^ layer (g kg^−1^), *Ti* is the soil thickness at the *i*^th^ layer (cm), *n* is the number of soil layers, and *pi* is the soil bulk density (g cm^−3^).

### Statistical Analyses

Linear regression and correlation analyses were performed with the SPSS 13.0 package for Windows. The least significant difference (LSD, *P* < 0.05) method was used to separate the means when differences were statistically significant. One-way ANOVAs followed by LSD multiple range tests were used for multiple comparisons of plant physiological and functional traits among the varying environmental factors. Canonical correlation analyses were used to test the relationships among plant features, leaf functional traits, soil characteristics, and environmental factors. The relationships among plant functional traits were tested by a Pearson correlation analysis. PCA analysis software online (http://www.metaboanalyst.ca) was also used to quantitatively determine the relationships among the related physiological traits and environmental factors of *C. korshinskii*.

## Additional Information

**How to cite this article**: Gong, C. *et al.* Carbon Storage Patterns of *Caragana korshinskii* in Areas of Reduced Environmental Moisture on the Loess Plateau, China. *Sci. Rep.* **6**, 28883; doi: 10.1038/srep28883 (2016).

## Supplementary Material

Supplementary Information

## Figures and Tables

**Table 1:** The biomass of four main organs in *Caragana korshinskii* along five experimental sites (n = 3 cover each organ).

**Sites**	**The biomass of different organs (kg m**^**−2**^)				
	**Branches**	**Leaves**	**Rachis**	**Roots**	**Total**
Yangling	0.33 ± 0.007^a^	0.09 ± 0.002^a^	0.02 ± 0.001^a^	0.20 ± 0.002^a^	0.64 ± 0.003^a^
Huangling	1.44 ± 0.015^c^	0.27 ± 0.003^b^	0.18 ± 0.002^b^	0.30 ± 0.003^b^	2.20 ± 0.006^b^
Ansai	1.01 ± 0.324^b^	0.84 ± 0.027^c^	0.29 ± 0.007^d^	0.31 ± 0.002^b^	2.45 ± 0.156^c^
Yulin	1.33 ± 0.021^c^	1.42 ± 0.026^d^	0.20 ± 0.003^c^	0.32 ± 0.004^b^	3.28 ± 0.012^d^
Shenmu	2.99 ± 0.042^d^	1.42 ± 0.020^e^	0.44 ± 0.006^e^	0.39 ± 0.001^bc^	5.24 ± 0.019^e^

Values represent mean ± se. Different letters indicate significant differences between sites studied (*P* ≤ 0.05).

**Table 2:** The relative contribution of organic distribution to the total organic carbon density in *Caragana korshinskii* (n = 3 cover each organic) and soil (n = 9) along 5 sites.

**Sites**	**The relative contribution of organic carbon density**				
	***Caragana korshinskii***				
	**Soil**	**Leaf**	**Rachis**	**Branch**	**Root**
Yangling	90.12 ± 0.95	1.33 ± 0.03	0.35 ± 0.01	5.28 ± 0.11	2.93 ± 0.08
Huangling	59.42 ± 0.62	4.99 ± 0.04	3.09 ± 0.05	28.62 ± 0.41	3.87 ± 1.65
Ansai	44.18 ± 0.79	18.64 ± 0.53	6.33 ± 0.65	23.62 ± 7.38	7.23 ± 0.22
Yulin	§7.09 ± 0.04	40.73 ± 0.91	5.44 ± 0.74	37.30 ± 0.32	9.42 ± 0.26
Shenmu	20.65 ± 0.18	21.55 ± 0.48	6.08 ± 0.33	45.93 ± 1.20	5.80 ± 0.03

Values are the mean ± se. ^§^Soil depths range from 0 to 300 cm (all except Yulin from 0 to 100 cm).
