# Supplementary material for: Carbon Storage Patterns of Caragana korshinskii in Areas of Reduced Environmental Moisture on the Loess Plateau, China
Source: Sci Rep. 2016 Jul 14;6:28883. doi: 10.1038/srep28883 (PMC4944161; doi:10.1038/srep28883)
Supplement: Supplementary Information [file srep28883-s1.pdf]

Original research paper

Title

Carbon Storage Patterns of *Caragana korshinskii* in Areas of Reduced Environmental Moisture on the Loess Plateau, China

Author names and affiliations

Chunmei Gong[1*], Juan Bai[1], Junhui Wang[1], Yulu Zhou[1], Tai Kang[1], Jiajia Wang[1], Congxia Hu[1], Hongbo Guo[1], Peilei Chen[2], Pei Xie[2], Yuanfeng Li[1]

[1]College of Life Sciences, Northwest A&F University, China.

Postal address: College of Life Sciences, Northwest A&F University, No.22 Xinong Road, Yangling, Shaanxi 712100, China.

Email address: Juan Bai (baijuan@nwsuaf.edu.cn), Junhui Wang (809934447@qq.com), Yulu Zhou (774801287@qq.com), Tai Kang (635351091@qq.com), Jiajia Wang (846563931@qq.com), Congxia Hu (627463533@qq.com), Hongbo Guo (127853980@qq.com), Yuanfeng Li (127858600@qq.com)

[2]College of Life Sciences, Zhejiang University, China.

Postal address: College of Life Sciences, Zhejiang University, No.866 Yuhangtang Road, Hangzhou, Zhejiang 310058, China.

Email address: Peilei Chen (921463383@qq.com), Pei Xie (963070580@qq.com)

[*]*Corresponding Author*

Chunmei Gong

[1]College of Life Sciences, Northwest A&F University, China.

Postal address: College of Life Sciences, Northwest A&F University, No.22 Xinong Road, Yangling, Shaanxi 712100, China.

Email address: gcm228@ nwsuaf.edu.cn

Work telephone number / Work fax number: 86 29 87092262

Supplementary information

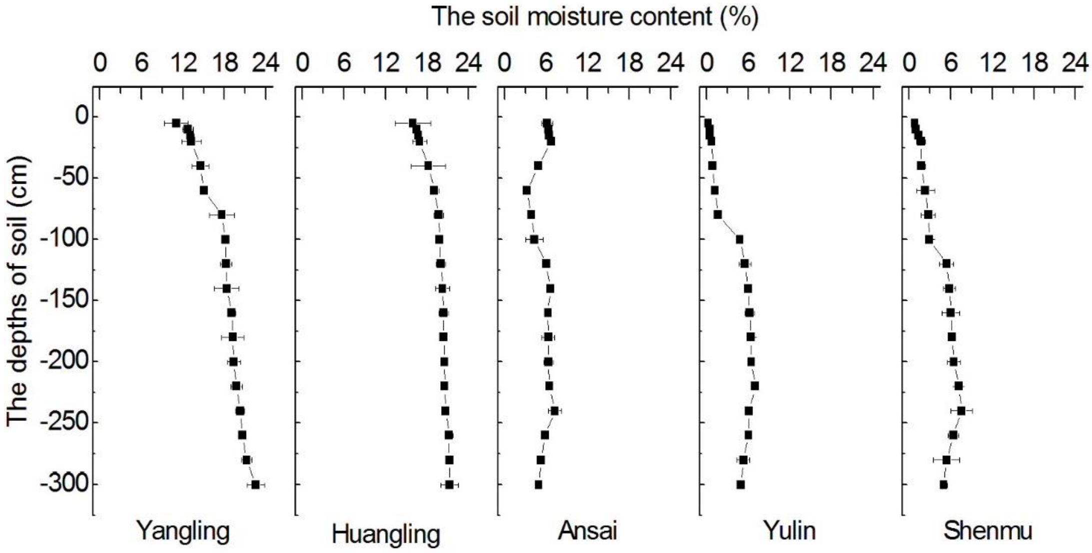

Supplementary Figure S1. Changes in soil water content with soil depth at the five experimental sites with reduced precipitation. Values are the mean ± se.

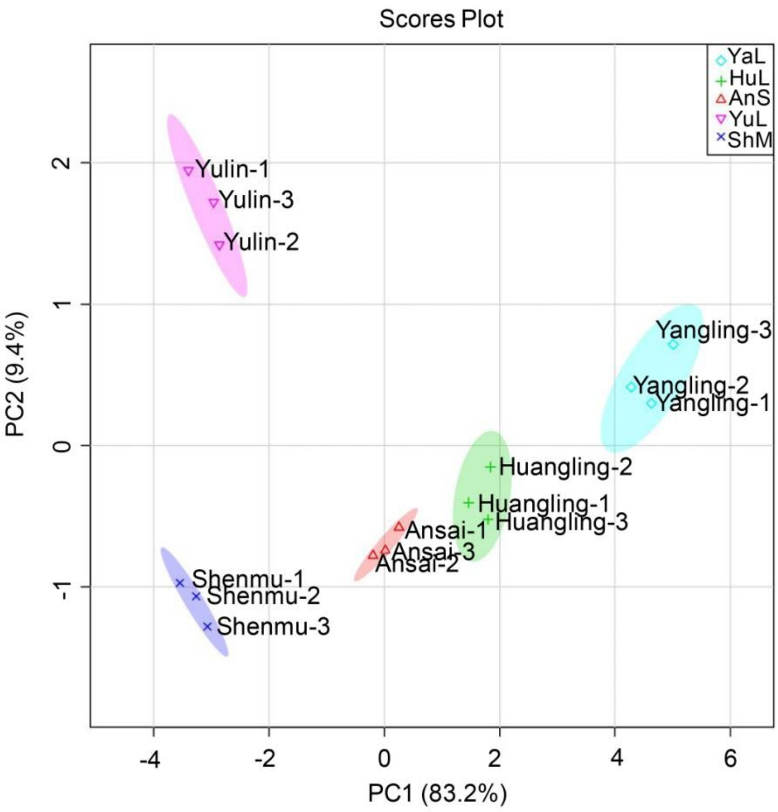

Supplementary Figure S2. PCA score plot showing variations in the environmental factors and functional traits at the five sites, which ranged from wet to dry. The first two PCs explain 92.6% of the total variability (83.2% (PC1) and 9.4% (PC2)). Yangling, Huangling, Ansai, and Yulin are explained by PC1, and Yulin and Shenmu are explained by PC2.

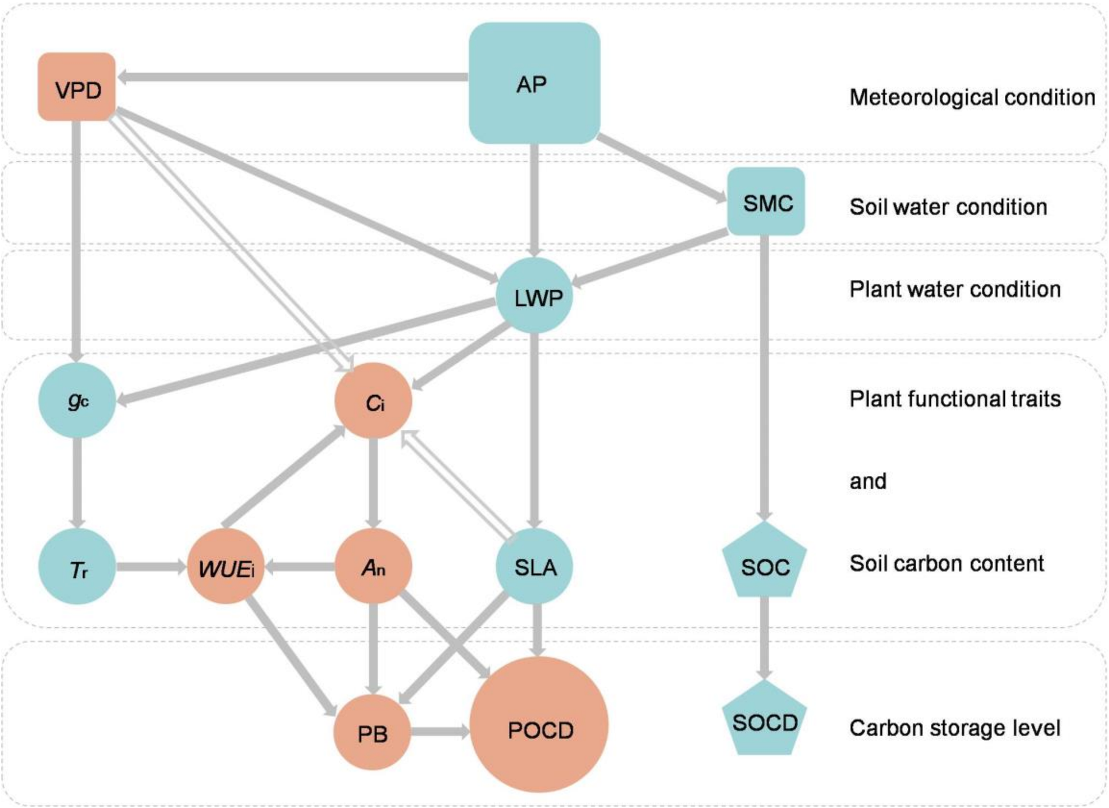

Supplementary Figure S3. Model of carbon sequestration and biomass for *Caragana korshinskii* under severe drought stress based on a correlation analysis. The leaf water potential (LWP) was most sensitive to decreased average annual precipitation (AP), leaf-air vapour pressure deficits (VPD), and soil moisture content (SMC). Water stress improved the mesophyll activity for intercellular CO_2 concentration (C_i) diffusion and then promoted net CO_2 assimilation rates (A_n) and inhibited canopy stomatal conductance (g_c) and transpiration (T_r), thereby instantaneously enhancing the water use efficiency (WUE_i), which promoted plant biomass (PB) and plant organic carbon density (POCD).

Note: Orange marks indicate increasing arid air stress and improved physiological parameters, and blue marks indicate increased environmental stress and decreased physiological indices with increased drought. Close arrows implicate the causal relationships of features traits and environmental factors was guided and derived with our results, and open arrows suggest it were quoted from others papers.

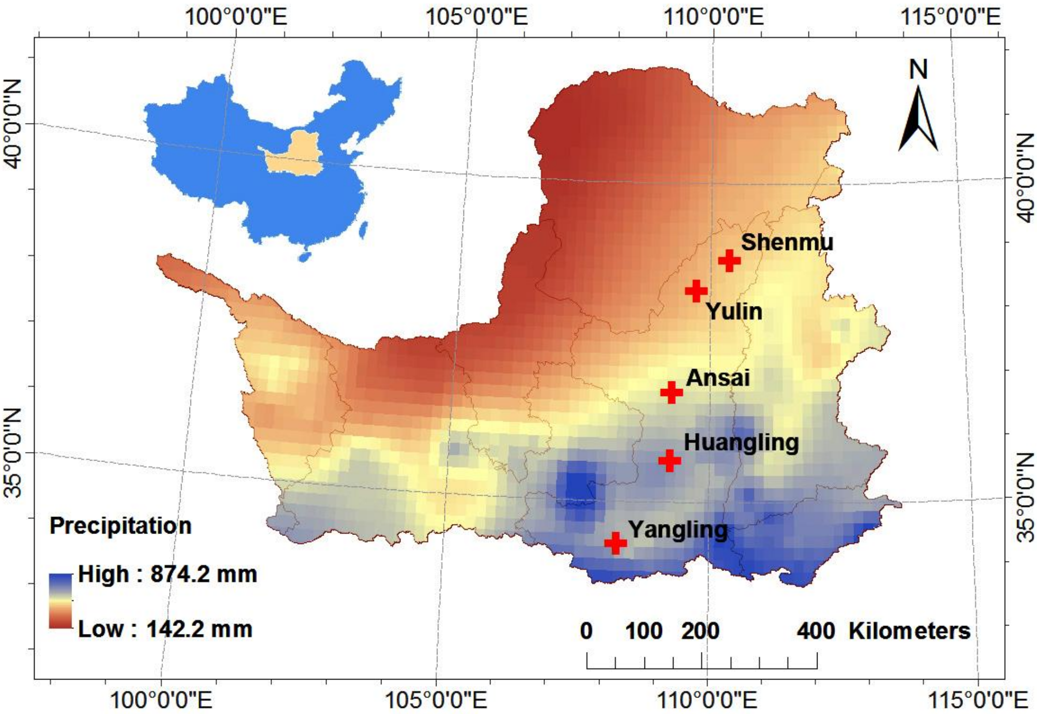

Supplementary Figure S4. Five experimental sites with reduced precipitation from south (Yangling) to north (Shenmu) along the Loess Plateau, northwestern China; these sites were selected to investigate the carbon sequestration of *Caragana korshinskii.* The map was created with the precipitation data during 1981-2010 acquired from the website of China Meteorological Data (http://data.cma.cn) using the ArcGIS software (the version number is ArcGIS 10.1) including ArcMap (http://www.arcgis.com/home). Five experimental sites were plotted in the map according to the longitude and latitude of each site we got using Global Position System previously.

	AP	AI	VPD	LWP	A_n	C_i	g_c	WUE_i	SLA	PB	POCD	SOC	SOCD	SMC
AP	1													
AI	-0.937**	1												
VPD	-0.970**	0.993**	1											
LWP	0.968**	-0.903*	-0.942**	1										
A_n	-0.904*	0.763	0.833*	-0.931*	1									
C_i	-0.963**	0.938**	0.965**	-0.910*	0.894*	1								
g_c	0.982**	-0.963**	-0.987**	0.958**	-0.905*	-0.988**	1							
WUE_i	-0.906*	0.990**	0.974**	-0.897*	0.717	0.885*	-0.930*	1						
SLA	0.862*	-0.731	-0.786	0.943**	-0.874*	-0.731	0.810*	-0.747	1					
PB	-0.923*	0.976**	0.966**	-0.910*	0.717	0.866*	-0.919*	0.989**	-0.794	1				
POCD	-0.915*	0.971**	0.961**	-0.907*	0.709	0.855*	-0.911*	0.989**	-0.796	1**	1			
SOC	0.947**	-0.792	-0.851*	0.945**	-0.907*	-0.843*	0.880*	-0.768	0.943**	-0.821*	-0.815*	1		
SOCD	0.955**	-0.800	-0.859*	0.930*	-0.901*	-0.865*	0.889*	-0.766	0.907*	-0.818*	-0.809*	0.995**	1	
SMC	0.998**	-0.913*	-0.953**	0.963**	-0.917*	-0.957**	0.972**	-0.876*	0.867*	-0.897*	-0.888*	0.960**	0.970**	1

Supplementary Table S1. Pearson correlation between environmental factors and functional traits of *Caragana korshinskii* with extremely significant negative correlation between average annual precipitation (AP), arid index (AI), leaf-air vapour pressure deficit (VPD), and soil moisture content (SMC). Plant functional traits were significantly affected by AP and SMC. Leaf water potential (LWP) was the most sensitive to AP, VPD, and SMC. Correlation between plant biomass (PB) and plant organic carbon density (POCD) was very high, and both were significantly related to instantaneous water use efficiency (WUE_i). * Correlation significant at the $P<0.05$ level, ** correlation significant at the $P<0.01$ level.

Trial sites	Location		Average annual precipitation (mm)	Arid index	Average annual temperature (℃)
Yangling	34°20'N	108°15'E	653.0 ± 9.0^{a}	1.52 ± 0.02^{d}	11.5 ± 0.3^{a}
Huangling	35°39'N	109°14'E	578.7 ± 8.8^{ab}	1.97 ± 0.18^{cd}	9.2 ± 0.1^{b}
Ansai	36°41'N	109°16'E	514.8 ± 12.3^{b}	2.74 ± 0.38^{bc}	9.0 ± 0.2^{b}
Yulin	38°19'N	109°50'E	435.1 ± 25.6^{c}	3.38 ± 0.38^{b}	10.0 ± 0.5^{ab}
Shenmu	38°47'N	110°21'E	419.1 ± 19.6^{c}	4.52 ± 0.13^{a}	8.7 ± 0.2^{b}

Supplementary Table S2. Geographical and meteorological profiles of five experimental sites chosen in this study. Our experimental sites are almost located in natural vegetation areas on the Loess Plateau. Meteorological data from the meteorological stations may not accurately reflect the actual precipitation because the experimental sites are likely to be far away from the nearest meteorological stations. Based on meteorological data from China Meteorological Data (http://data.cma.cn) and Ecological Environment Database of Loess Plateau (http://www.loess.csdb.cn/pdmp/index.action), we amended environmental parameters of each site by referring to related literatures and the Internet, and acquired environmental backgrounds of the most close to vegetation growth years of the experimental sites. Values are the mean ± se. Letters indicate significant differences between sites (P<0.05).

Supplementary information accompanies this paper at http://www.nature.com/srep
